# Environmental selection is a main driver of divergence in house sparrows (*Passer domesticus*) in Romania and Bulgaria

**DOI:** 10.1002/ece3.2509

**Published:** 2016-10-11

**Authors:** Julia C. Geue, Csongor I. Vágási, Mona Schweizer, Péter L. Pap, Henri A. Thomassen

**Affiliations:** ^1^ Comparative ZoologyInstitute for Evolution and EcologyUniversity of TübingenTübingenGermany; ^2^ MTA‐DE ‘Lendület’ Behavioural Ecology Research GroupDepartment of Evolutionary Zoology and Human BiologyUniversity of DebrecenDebrecenHungary; ^3^ Evolutionary Ecology GroupHungarian Department of Biology and EcologyBabeş‐Bolyai UniversityCluj‐ NapocaRomania; ^4^ Animal Physiological EcologyInstitute for Evolution and EcologyUniversity of TübingenTübingenGermany

**Keywords:** Eastern Europe, evolutionary process, isolation by adaptation, isolation by distance, landscape genetics, *Passer domesticus*

## Abstract

Both neutral and adaptive evolutionary processes can cause population divergence, but their relative contributions remain unclear. We investigated the roles of these processes in population divergence in house sparrows (*Passer domesticus*) from Romania and Bulgaria, regions characterized by high landscape heterogeneity compared to Western Europe. We asked whether morphological divergence, complemented with genetic data in this human commensal species, was best explained by environmental variation, geographic distance, or landscape resistance—the effort it takes for an individual to disperse from one location to the other—caused by either natural or anthropogenic barriers. Using generalized dissimilarity modeling, a matrix regression technique that fits biotic beta diversity to both environmental predictors and geographic distance, we found that a small set of climate and vegetation variables explained up to ~30% of the observed divergence, whereas geographic and resistance distances played much lesser roles. Our results are consistent with signals of selection on morphological traits and of isolation by adaptation in genetic markers, suggesting that selection by natural environmental conditions shapes population divergence in house sparrows. Our study thus contributes to a growing body of evidence that adaptive evolution may be a major driver of diversification.

## 1 Introduction

It has become clear through theoretical and empirical research that neutral as well as selective evolutionary processes can result in population divergence and ultimately lead to speciation (e.g., Coyne & Orr, 2004). While neutral processes such as isolation by dispersal limitation (IBDL; Orsini, Vanoverbeke, Swillen, Mergeay, & De Meester, 2013) can lead to a pattern of isolation by distance (IBD; Wright, 1943) or isolation by landscape resistance (McRae, 2006), it is unclear how influential these forces are, and recent evidence suggests instead that divergent selection may be a major driver of evolutionary change (e.g., Ellner, Geber, & Hairston, 2011; Hendry & Kinnison, 2001). Currently, the relative importance of each of these processes often remains unresolved (Mitchell‐Olds, Willis, & Goldstein, 2007).

Both neutral and selective processes have been well studied and documented (e.g., Mitchell‐Olds et al., 2007), but have in many cases been investigated independently from one another. However, it is crucial to simultaneously assess the potential role of neutral divergence and that of selection in a comparative framework. Classic approaches to demonstrate the presence of selection and local adaptation in a species are common garden or reciprocal transplant experiments in which the fitness of individuals from locations with strong environmental differences are compared (Kawecki & Ebert, 2004). Advantages to this approach include the acquisition of direct evidence for local adaptation and the potential to quantify the resulting fitness consequences to then identify the specific agent of selection (Kawecki & Ebert, 2004). However, such experiments are difficult to apply to organisms with long generation times, complex ecological requirements, or life cycles that are difficult to mimic experimentally (Savolainen, Lascoux, & Merila, 2013).

As an alternative, landscape genetic approaches directly associate phenotypes or genotypes with environmental variables and measures of geographic distance or topography (Manel, Schwartz, Luikart, & Taberlet, 2003; Storfer et al., 2007). While these approaches do not provide fitness estimates, their power lies within the joint processing of biological traits and a large variety of environmental variables measured on the ground and from remote sensors. To this end, morphological measurements are useful markers as they may directly represent responses to natural selection. However, whether or not such a response has an adaptive genetic basis, or is merely plastic, remains unclear. To complement morphological measurements, as a genetic marker of choice, easily obtained microsatellite repeat markers do not provide insight into specific adaptations, but are nevertheless useful in a first‐order assessment of the overall relative importance of neutral and selective processes in driving and maintaining population divergence (Orsini et al., 2013). Such neutral markers diverge through the process of genetic drift, effects of which are maintained by either increasing geographic distance, physical barriers or inhospitable habitat conditions between populations (landscape resistance; McRae, 2006), or by the reduced fitness of dispersing individuals that are maladapted to the conditions at new locations (Nosil, Funk, & Ortiz‐Barrientos, 2009). Thus, a correlation between neutral markers and environmental variables that cannot be explained by geographic distance alone may be indicative of divergent selection driving population divergence, a phenomenon termed isolation by adaptation (IBA; Nosil et al., 2009).

House sparrows (*Passer domesticus*) are a suitable species to examine landscape‐level patterns of intraspecific variation, because they are widespread and occur along a range of different environmental conditions that may pose divergent selection pressures (Kekkonen, Seppa et al., 2011; MacGregor‐Fors, Morales‐Pérez, Quesada, & Schondube, 2010; Vangestel et al., 2012). Here, we studied the relative roles of neutral and selective processes on the divergence of natural house sparrow populations in Romania and Bulgaria, a still understudied region in Europe. To do so, we: (1) analyze the population genetic structure based on microsatellite markers; (2) relate morphological and genetic variation to environmental variables and measures of geographic distance and landscape resistance; and (3) compare the importance of natural habitat variables with those related to human habitation. Finally, because protecting standing intraspecific variation will help maximizing a species’ evolutionary potential facing changing environmental conditions (Brooks et al., 2015; Dawson, Jackson, House, Prentice, & Mace, 2011; Frankham, 2010; Grivet, Sork, Westfall, & Davis, 2008; Hartl, Zachos, & Nadlinger, 2003; Matala, Ackerman, Campbell, & Narum, 2014; Smith et al., 2001; Thomassen et al., 2011; Vandergast, Bohonak, Hathaway, Boys, & Fisher, 2008), and intraspecific variation in common species may represent that in species of conservation concern (e.g., Thomassen et al., 2011), we also aimed to map intraspecific variation in house sparrows in Romania and Bulgaria for conservation purposes. We used morphological and genetic data collected from 691 individuals from 33 populations distributed across and along environmental gradients in temperature, precipitation, elevation, and land cover. As morphological markers, we used the size and shape components resulting from a “PCA ratio spectrum” analysis (Baur & Leuenberger, 2011) of a set of measurements describing primarily wing, tail, and tarsus sizes. We complemented our morphological dataset with twelve microsatellite markers, eight of which were found to be polymorphic. To then relate intraspecific variation to environmental variables, we used a dissimilarity‐based matrix regression (generalized dissimilarity modeling; GDM) technique that—in contrast to other methods often applied—can simultaneously take into account the effects of distance and environment (Ferrier, Manion, Elith, & Richardson, 2007).

## 2 Methods

### 2.1 Study region

**Figure 1:**
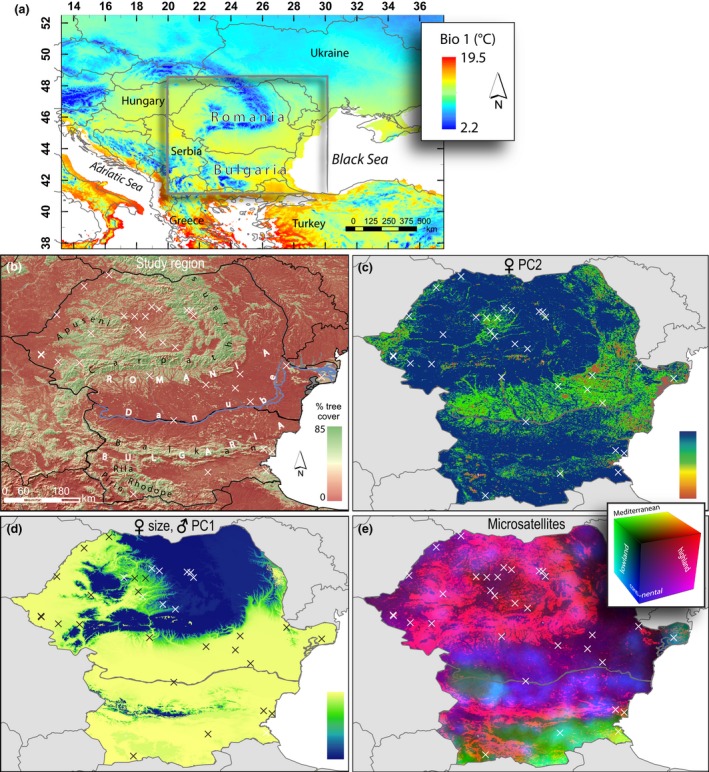
Study region, sampling sites, and generalized dissimilarity modeling results. (a) Location of the study region within Eastern Europe, with average temperature of the year (Bio 1). (b) Overview of the study area, with sampling sites (crosses) on a hillshade map and an overlay of percent tree cover. (c–e) GDM results for the second morphological shape component for females (c), the morphological size component in females and the first shape component for males (d), and microsatellites (e). The color difference between two locations along the color bar (c, d) or on the RGB color cube (e) in the GDM maps represents the magnitude of the difference in the biotic response variable, that is, morphological variable or *F*_ST_

Romania and Bulgaria are located in southeastern Europe (Figure 1a) and comprise distinct climatic zones: the continental and Mediterranean climatic zones in Bulgaria, and the continental and temperate climatic zones in Romania. The Danube River forms a natural border along much of its length between Romania in the north and Bulgaria in the south. Large mountainous areas, with peaks up to about 2,500 m, cover much of the land surface in these countries; in Romania, the Carpathian mountain region is predominant, whereas the Balkan, Rhodope, Rila, and Pirin mountains merge to a large mountainous area in Bulgaria (Figure 1b). At a smaller scale, the landscape in this region can be characterized as extensive and intensive agriculture interspersed with seminatural areas consisting of forest, open woodland, and grassland. As a result of this variation of habitats, different biogeographical regions are recognized, including the continental, alpine, steppic, black sea, and pannonian regions (Council of Europe (CoE) 2015). This habitat mosaic constitutes an ideal test bed to study evolutionary processes in natural populations, because of its high habitat heterogeneity across short distances, allowing for the potential of strong divergent selection pressures on natural populations.

### 2.2 Study species

House sparrows are a widespread, synanthropic species (Anderson, 2006). It has been suggested that factors related to human habitation and land use play a key role in the abundance and genetic diversity of house sparrow populations (Kekkonen, Hanski, Jensen, Väisänen, & Brommer, 2011; Vangestel et al., 2012). Postnatal dispersal distances are low, ranging between 1 and 1.7 km (Anderson, 2006; Paradis, Baillie, Sutherland, & Gregory, 1998), allowing for the potential for population divergence to be driven by IBD (Kekkonen, Hanski et al., 2011; Vangestel et al., 2012). Previous studies of house sparrow population structure in other regions demonstrated varying levels of divergence. For instance, Finnish populations were found to be essentially panmictic, with little evidence for a pattern of IBD (Kekkonen, Seppa et al., 2011). In contrast, populations in mainland Norway and associated islands showed low‐to‐moderate divergence, most likely caused by IBD (Jensen et al., 2013). Similarly, weak but significant structure was observed in native populations in Belgium (Vangestel et al., 2012) and France (Liu et al., 2013), and in introduced populations in Brazil (Lima et al., 2012).

### 2.3 Field sampling

Samples were collected in 2007 and 2008, and 2013–2015 at 33 locations throughout Romania and Bulgaria (Figure 1b; Table S1). Sites were selected based on two key criteria: (1) the full set of sites covers as much as possible of the environmental niche breadth observed in the study area and (2) sites are located across as well as along environmental gradients, such that the potential effects of geographic distance and environmental gradients on population divergence are decoupled and can be distinguished in subsequent correlative analyses. Identification of gradients and selection of sites were performed using available climate and satellite remotely sensed habitat data at 0.25‐ to 1‐km resolutions (see below). All sampling locations were near anthropogenic sites. Birds were captured using mist nets, which were set up around villages and at the edges of gardens or farms. Individuals were sexed, morphological measurements were recorded, and DNA samples obtained via two tail feather and blood samples. Feathers were stored dry in envelopes and blood samples in >96% ethanol. Birds were banded and released immediately after processing at the site of their capture. In total, 691 individuals were sampled (on average ~ 21 per site): 314 males, 302 females, and 75 unsexed individuals.

### 2.4 Morphological measurements and analyses

Because samples were collected over the course of several years and additional measurements were added later in this study, a set of three morphological measurements were available for all individuals (wing length, tail length, and tarsus length), and an additional four measurements for only a subset of our samples (populations from Ognyanovo, Beli bryag, Jasna poljana, Popovits, Golica, Poiana, Berzovia, Salonta, Parta, Caransebeş, Mihăeşti, Hălmagiu, Măgheru__, Runc and Lăzarea): culmen length, bill depth, head width, and head length. Morphological data were analyzed for adult individuals only, and because of sexual dimorphism in this species (with males being generally larger than females), for males and females independently.

Raw morphological measures are unlikely to be independent from one another due to allometric relationships, and as a result, we used the raw morphological data to create a size and several independent shape components using the “PCA ratio spectrum” method developed by Baur and Leuenberger (2011) (Appendix S1). For the size component and each shape component that explained >10% of the total variation in the PCA, we computed population pairwise differences as follows: $\left|\overset{¯}{x}-\overset{¯}{y}\right|/\sigma_{x}+\sigma_{y}$, where $\overset{¯}{x}$ and $\overset{¯}{y}$ are the averages for populations x and y and $\sigma_{\overset{¯}{x}}$ and $\sigma_{\overset{¯}{y}}$ are their standard deviations. Because we only had partial datasets—one with three morphological variables (wing, tail, and tarsus lengths) for all locations, and one with all morphological variables (also including culmen and head lengths, head width, and bill depth) for only nine locations, we evaluated which one was the most appropriate to use. Our assessment suggested that the three‐variables‐all‐locations dataset gave the most robust results (Appendix S1).

### 2.5 Laboratory methods and genotyping

DNA was extracted using the QIAGEN DNeasy Blood & Tissue Kit (Qiagen, Hilden, Germany) following the manufacturer's protocol. Because of potential misidentification of Spanish sparrows (*P. hispaniolensis*) as house sparrows, we genetically identified individuals to species from southern sampling sites using the cytochrome c oxidase subunit I (COI) mitochondrial gene (Appendix S1).

We genotyped house sparrow individuals for twelve published microsatellite loci (Dawson et al., 2012; Garnier et al., 2009; Griffith et al., 2007) (Table S2). Of these twelve loci, two were monomorphic in the majority of the sampling locations after initial genotyping of a subset of individuals and omitted from further analyses. Fragment length analysis was carried out on an ABI 3730 sequencer at the University of Turku, Finland. Results were analyzed with GeneMarker V2.4.1 (Softgenetics, State College, PA, USA).

### 2.6 Population genetic analyses

Because only a few birds could be sampled at certain locations, we calculated the geographic distances between locations, and pooled locations with small sampling sizes with those nearby (Măgheru__ and Runc were 13.8 km apart; Parta 1 and Parta 2 3.6 km). Loci were checked for the presence of null alleles using MICRO‐CHECKER (Van Oosterhout, Hutchinson, Wills, & Shipley, 2004), deviations from Hardy–Weinberg Equilibrium (HWE) using GenAlEx 6.501 (Peakall & Smouse, 2012), and Linkage Disequilibrium (LD) using GENEPOP web version 4.2 (Rousset, 2008). We used COLONY version 2.0.5.9 to identify full siblings within sampling sites. Presence of full siblings would confound Bayesian clustering analyses and *F*_ST_ estimates. All individuals were coded as offspring. No full‐sib ships were detected (results not shown); hence, all individuals were kept for subsequent analyses. To assess the level of genetic structure, we conducted two Bayesian clustering analyses: STRUCTURE (Pritchard, Stephens, & Donnelly, 2000) and GENELAND (Guillot, Estoup, Mortier, & Cosson, 2005; Guillot, Mortier, & Estoup, 2005; Guillot, Santos, & Estoup, 2008) (Appendix S1).

For subsequent landscape genetic analyses, we calculated site pairwise *F*_ST_ values. Because a signal of null alleles was detected, we computed corrected *F*_ST_ using the “excluding null alleles” (ENA) method implemented in FreeNA with 10,000 bootstrap replicates (Chapuis & Estoup, 2007). To minimize the risk that potential correlations between *F*_ST_ and environmental variables are the result of demographic processes, we evaluated whether or not population divergence was simply a result of differences in genetic variation within populations (Appendix S1). We also tested whether morphological divergence and genetic population divergence were concordant using a Mantel test with 999 permutations.

### 2.7 Environmental variables

To describe environmental conditions across Romania and Bulgaria, we compiled a set of 34 environmental variables related to climate, topography, vegetation, and human habitation (Table S3) at 30 arcsec resolution. Although the home‐range sizes of individual birds are likely much smaller, spatial heterogeneity in climate variables within each grid cell is small compared to that between distant grid cells, and dispersal has been reported to be up to 1.7 km (Anderson, 2006; Paradis et al., 1998). The used spatial resolution of variables thus balances home‐range size with dispersal distances as well as availability and computational tractability of subsequent analyses. Bioclimatic variables expressing variations in temperature and precipitation were obtained from WorldClim (http://www.worldclim.org/) (Hijmans, Cameron, Parra, Jones, & Jarvis, 2005). These variables are derived from a network of weather stations and are based on a 50‐year climatology from 1950 to 2000. Elevation data were obtained from the Shuttle Radar Topography Mission (SRTM) and used directly in further analyses as well as to compute slope (steepness of the terrain) and aspect (the compass direction that a slope faces). Vegetation data included the percent tree cover from 2001 (Hansen et al., 2002) and Leaf Area Index (LAI; Myneni et al., 2002) obtained from the Global Land Cover facility database (http://www.glcf.umd.edu/data/). We also used a measure of surface moisture based on the QuikSCAT microwave instrument (QSCAT; Long, Drinkwater, Holt, Saatchi, & Bertoia, 2001). For areas with dense forest, QSCAT is sensitive to canopy roughness. We computed multiyear (2000___2008) averages of raw backscatter measurements at the horizontal polarization, including means, minima, maxima, and seasonality (expressed as the coefficient of variation). Further details on the computation of QSCAT variables are provided in Appendix S1.

Because sparrows are commensal with anthropogenic activity, we also included two measures of human habitation as predictors: road density and human population density. The road density layer was created out of a shape file of roads (Digital Chart of the World, downloaded from http://www.diva-gis.org/gdata on 21 November 2013), processed in ArcGIS 10.0 (ESRI, Redlands, USA) using the “line density tool.” The output cell size was set to 0.0083333 degrees (i.e., 30 arcsec) to match the other environmental variables, and because of the short natal dispersal and small home‐range sizes of sparrows (Anderson, 2006; Paradis et al., 1998), the search radius was set to five map units (~5 km). Human population density data were obtained from the Gridded Population of the World dataset, version 3 for the year 2000 at 2.5 arcmin (~5 km) resolution (Center for International Earth Science Information Network (CIESIN), Columbia University 2005; http://sedac.ciesin.columbia.edu retrieved 12 May 2015).

In addition to straight‐line geographic distance, we included two other types of distance that may be more realistic measures of the distance dispersing individuals have to travel to reach another location. First, because of the short postnatal dispersal distances of house sparrows (1–1.7 km; Anderson, 2006; Paradis et al., 1998) and the width of the Danube River at places reaching 1.5 km, the Danube was included as a barrier to dispersal. In a GIS layer, areas north of the Danube River were coded 0, and those south 1, resulting in differences of 1 between sampling sites across the river and of 0 between those on one side of the river. Second, we computed resistance distances based on human population density in Circuitscape 3.5.8 (McRae, 2006). To do so, human population density was treated as a conductance map (i.e., higher densities are favorable to dispersal and gene flow), and a cell connection scheme of eight neighbors was used.

To reduce this set of environmental variables to a smaller suite that each provided unique information, we extracted their values at the sampling sites using ArcMap 10.2.2 (ESRI, Redlands, USA) and computed Pearson correlation coefficients (logistic regression in the case of the Danube River barrier) in R 3.1.2 (Table S4). When two variables had a Pearson correlation coefficient ≥0.7 (or *p* < .05 for the logistic regression), one of them was excluded from further analyses. Of those pairs, we retained the one that is more easily interpretable (e.g., Bio 1: mean temperature of the year versus Bio 3: isothermality).

### 2.8 Landscape genetic analyses

To assess correlations of morphological or genetic data with environmental variables, geographic distance, and landscape resistance, we used generalized dissimilarity modeling (GDM; Ferrier et al., 2007), implemented in the R package gdm (Manion, Lisk, Ferrier, Neito‐Lugilde, & Fitzpatrick, 2015). GDM has increasingly been used in landscape genetic studies, including in tests for IBA (Freedman, Thomassen, Buermann, & Smith, 2010; Mitchell, Locatelli, Sesink Clee, Thomassen, & Gonder, 2015; Thomassen et al., 2010). It is an iterative matrix regression method that fits dissimilarities of predictor variables to dissimilarities of a response variable. It can analyze and predict spatial patterns of beta diversity across large areas, using I‐spline basis functions to adjust nonlinear relationships between environmental variables and biological variation (Ferrier et al., 2007). In this work, GDM was used to predict the relationship between a set of predictor variables and pairwise genetic distances (*F*_ST_) or morphological differences as response variables. The predictor variables consisted of environmental variables, geographic distance, resistance distance, and the Danube barrier, and were selected to determine the biotic variation that is explained by IBA, isolation by distance (IBD; Wright, 1943), or resistance by the habitat matrix in between populations. The importance of predictor variables is tested by permutations, where only variables that contribute significantly to explaining variation in the response variable are retained. The relative importance of predictor variables can be evaluated by examining the maximum height that is reached in variable response curves. In total, five types of models were performed: (1) a best fit model (including all environmental variables, as well as geographic distance); (2) a model with only the environmental variables; (3) a model with only straight‐line geographic distance; (4) a model with only resistance distances; and (5) a set of 1,000 models with random environmental variables to evaluate the significance of the variation explained by the best fit model. The best fit model was considered not significant if the variation explained fell below the upper 95% confidence interval of the random models. Model fit was visualized in a scatter plot of predicted versus observed response values.

In a subsequent step, the spatial distribution of the response variable can be projected across the study area using the known environmental conditions (obtained from the predictor variables) outside the sampling locations and the calculated relationship between the environment and biological variation. We visualized this variation in the response variables in three‐dimensional RGB color space. To do so, we followed the computationally tractable approach from Fitzpatrick and Keller (2015). Briefly, we first extracted the values of the retained environmental variables at a grid with 30 arcsec resolution, corresponding to the midpoints of grid cells in the 30 arcsec WorldClim dataset. We then “transformed” the environmental variables for these sites into a set of “genetic importance” variables (Fitzpatrick & Keller, 2015). We conducted a principal component analysis (PCA) on these transformed variables to obtain a smaller set of independent variables. We then matched RGB values to the first three PC axes, which were subsequently combined into one multiband RGB GIS layer in ArcMap 10.2.2 (ESRI, Redlands, USA). We verified that the resulting maps were concordant with those obtained using the “predict.gdm” function with subsequent multidimensional scaling, but which was only possibly at low resolution due to computational limitations (Appendix S1).

Finally, to inform conservation practices in Romania and Bulgaria, we visually assessed whether current protected areas capture genetic and morphological variation in house sparrows sufficiently well (Appendix S1).

## 3 Results

### 3.1 Size and shape components of morphological measurements

We used the “PCA ratio spectrum” method to distinguish between the size and shape components of the morphological measurements. PCA results of the shape component are shown in Table S5. For both males and females, the first two extracted principal shape components explained all of the observed variation. PCA ratio spectra for wing, tail, and tarsus length are nearly identical for males and females (Figure S1) and suggest that most variation along the first axis is explained by the ratio of tail and tarsus, and along the second axis by the ratio of tail and wing. These results for the dataset with just wing, tail, and tarsus length but for all locations are supported by those for the all‐variables‐nine‐locations dataset for the first axis. This was, however, not the case for the second axis, where tail and wing are close to one another on the axis (explaining very little of the variation), bill depth is positioned on one end of the spectrum, contrasted on the other end by tarsus and culmen in males and by tarsus and head measures in females. Along the third axis, most variation is explained by the ratio between culmen and tail in both males and females, but bill depth is also important in males, whereas it is not in females. We conducted subsequent landscape genetic analyses using the PC scores of the size component and the first two shape components in males and females separately.

### 3.2 Population genetic analyses

The number of effective alleles (*N*_*E*_) ranged from 3.184 to 6.887; *H*_*O*_ from 0.583 to 0.846; and *H*_*E*_ from 0.590 to 0.794 (Table S6). Two microsatellite loci were found to be out of HWE in many sampling locations: Pdo31 significantly deviated from HWE in 15 locations and Pdo7 in 25 locations. These loci were, therefore, omitted from further analyses. After Bonferroni correction, no loci were in significant LD. We found a signal for the presence of null alleles and therefore calculated ENA‐corrected (Chapuis & Estoup, 2007) *F*_ST_ values for the remaining eight loci to be used in subsequent landscape genetic analyses. The global population variation across all loci and all sites was *F*_ST_ = 0.011.

STRUCTURE analyses using the admixture model with location prior and either correlated or noncorrelated allele frequencies suggested there is no clear genetic structuring among sparrow populations in Romania and Bulgaria (*K *=* *1). Inclusion of the spatial component using GENELAND supported this finding. When we did not use a model for null alleles, all ten independent runs inferred six clusters (*K *=* *6). However, assignments of cluster membership were highly inconsistent between runs (not shown), and we therefore concluded that there was little evidence for significant population structure based on these analyses. A lack of clear population genetic structure, however, does not necessarily mean a lack of IBD or IBA; merely that selection pressures may be relatively low, or there is a much relatively recent or ongoing gene flow. In fact, correlation analyses between genetic divergence and environmental heterogeneity may be better suited to identify potential patterns of IBD or IBA than those purely based on genetic data. We, therefore, proceeded with landscape genetic analyses using the ENA‐corrected *F*_ST_ values (Chapuis & Estoup, 2007).

Mantel tests between *F*_ST_ and morphological divergence were only significant for shape PC2 in females but with a low correlation (*Z* = 23.80624, *r* = .289, *p* = .001 for 999 permutations; for female size *Z* = 17.79662, *r* = .111, *p* = .063 for 999 permutations; for male PC1 *Z* = 24.01073, *r* = .137, *p* = .075 for 999 permutations).

### 3.3 Landscape genetic analyses

Among the morphological variables, models for the first shape component (shape PC1) in males and for the size and second shape (shape PC2) components in females performed better than random models (Table 1). For shape PC1 in males, geographic distance was included in the best fit model, but explained very little of the variation when used alone, and similar results were found for size and shape PC2 in females. Thus, IBD appears to play only a minor role in driving population divergence in morphological variables. This finding is supported by the lack of a correlation between morphological divergence and geographic distance (Figures S2c–S4c). The mean temperature of the driest quarter (Bio 9) was the most important variable explaining variation in PC1 for males and size in females (Figures S2a and S3a), whereas minimum leaf area index (LAImin) was the most important variable describing variation in shape PC2 in females (Figure S4a). Variables related to human habitat contributed little (for shape PC2 in females) to no explanatory power to help distinguish morphological variation.

**Table 1:** Results of generalized dissimilarity models of the size and shape components of wing, tail, and tarsus length measurements and of microsatellites

	Best fit	Env only	Dist only	Random	Lower CI	Upper CI
Males’ size	6.8	6.8	0.2	6.3	6.2	6.4
Males’ shape PC1	30.3	30.1	0.0	7.0	6.9	7.2
Males’ shape PC2	6.6	6.6	0.0	6.5	6.4	6.6
Females’ size	14.7	14.4	0.0	6.5	6.3	6.6
Females’ shape PC1	2.1	2.1	0.0	5.6	5.5	5.7
Females’ shape PC2	27.3	27.3	1.9	6.5	6.4	6.7
Microsatellites	25.0	25.0	3.8	4.4	4.3	4.4

Numbers represent the total observed variance (%) explained by the best fit model (Best fit) and models with only environmental variables (Env only), only geographic distance (Dist only), and the mean value of 1000 models with random environmental variables (Random) and the associated confidence intervals (Lower CI, Upper CI).

The best fit generalized dissimilarity model for microsatellites, where all variables were entered in the model, explained 24.95% of the observed variation (Table 1) and only retained environmental variables in the final model. A model with only geographic distance or resistance distance as the predictor variable explained 3.84% and 7.93% of the variation, respectively, and random models explained 4.35% of the variation, with an upper confidence level of 4.44%. These results also suggest that local environmental conditions rather than isolation by distance or isolation by resistance are important in generating house sparrow population genetic divergence, which is supported by a lack of correlation between *F*_ST_ and geographic distance (Figure S5c). The variables most important in explaining the observed genetic variation were annual precipitation (Bio 12), mean leaf area index (LAImean, a measure of greenness), mean temperature of the driest quarter (Bio 9), and precipitation of the driest month (Bio 14) (Figure S5a). Road density was also retained as an explanatory variable, but did not contribute as much as the above‐mentioned climate and vegetation variables.

## 4 Discussion

### 4.1 Landscape genetics

We examined whether neutral (isolation by dispersal limitation) or selective evolutionary processes are the most important drivers of house sparrow population divergence in Romania and Bulgaria and whether measures of human habitation play a role in the divergence in this human commensal species. We found that IBDL could not explain either morphological or genetic divergence, whereas environmental variables explained a large proportion (up to 30%) of the observed variation. Our results for morphological measurements were thus consistent with a signal of selection. Although the number of polymorphic microsatellite markers was relatively low, and a large set of SNP markers will be more suited to get insight into population divergence and selection at the genetic level, results for microsatellites were nevertheless consistent with a pattern of IBA, and thus support the morphological data in the notion that adaptive processes are more important than neutral ones in driving population divergence. Our results suggesting that divergent natural selection is a main driver of intraspecific variation in this species are in agreement with findings for populations in Norway (Holand, Jensen, Tufto, & Moe, 2011), Brazil (Lima et al., 2012), and France (Liu et al., 2013). However, these studies were conducted at much smaller scales, with much fewer populations. Moreover, those in Norway and Brazil did not relate population divergence to environmental variables, but rather compared *F*_ST_ to estimates of morphological divergence (*Q*_ST_ or *P*_ST_). Perhaps more importantly, the study in France found fine‐scale spatial autocorrelation, suggesting IBDL at short distances, but the potential effect of distance was not included in subsequent correlative analyses with environmental factors, making it difficult to assess the relative importance of IBDL versus IBA.

The spatial patterns of morphological variation in the size component in females and shape PC1 in males show a very sharp division between higher and lower elevation areas (Figure 1d) due to a large response to small differences in mean temperature of the driest quarter (Bio 9) between mountain and lowland areas, which then levels off to a flat response at larger differences (Figures S2a and S3a). In contrast, the spatial pattern of variation in shape PC2 in females is more complex (Figure 1c): the main turnover of the morphological measures occurs at smaller differences in minimum leaf area index (LAI min; Figure S4a). The potential underlying causal relationship between shape PC2 in females—dominated by wing length—and minimum leaf area index remains unclear. Wing length in birds is often related to vegetation density, where individuals from forests tend to have shorter wings than those from the open field because of the advantage of shorter wings for maneuverability in dense vegetation; however, we did not find such a relationship in our house sparrow samples (results not shown), nor did we find that leaf area index was an important factor in the shape components of males, as would be expected given that both males and females should exhibit similar selection pressures for wing length related to vegetation. As for microsatellite variation, spatial patterns roughly follow a lowland versus highland and Mediterranean versus continental subdivision (Figure 1e). Specifically, higher elevation populations are genetically similar, but lowland populations from southern Bulgaria, with a more Mediterranean climate, are distinct from those in Romania, where a more continental climate prevails. In addition, lowland populations from the Danube Delta are nearly as distinct from other lowland populations as the latter are from higher elevation populations.

Despite only subtle population differentiation at the genetic level, we found that divergence is tied to the environment, independent of geographic distance. Further support for these findings comes from visual inspection of observed versus predicted values and plots of population divergence versus geographic distance (Figures S2b–S5b and S2c–S5c, respectively). Of all variables entered into the models, only a small set was selected that explained most of the observed variation (Figures S2a___S5a), notably mean temperature of the driest quarter (Bio 9), annual precipitation (Bio 12), and mean and minimum leaf area index (LAI mean, LAI min). However, visual examination of the shape of the response curves suggests that the effects of those predictor variables vary between response variables. For instance, for the shape PC1 in males (Figure S2a) and the size component in females (Figure S3a), there is a very steep response to small changes in the mean temperature of the driest quarter (Bio 9), which then quickly levels off. In contrast, for genetic variation, small differences in the mean temperature of the driest quarter (Bio 9) do not result in larger *F*_ST_ values (Figure S5a); larger differences, however, result in exponentially increasing divergence. These results thus suggest that divergence in morphological traits is not shaped by the same environmental variables as in microsatellites. Further insight into this issue comes from the correlations between *F*_ST_ and morphological divergence, as well as from a comparison of the spatial patterns of variation shown in the GDM maps (Figures 1c–e). A crude subdivision into highland and lowland populations in both morphological traits and microsatellites and a small but significant correlation between *F*_ST_ and female shape PC2 suggest that similar factors may underlie population divergence in phenotype and genotype. However, finer substructuring of populations and a lack of correlations between *F*_ST_ and female size and male shape PC1 indicate that such a pattern is not broadly supported. Thus, if genetic divergence indeed is related to IBA, the factors that limit gene flow, leading to neutral divergence in microsatellites, must be primarily physiological characteristics or morphological variables other than those measured here.

Although in our study selective processes appeared to be the most important factors underlying population divergence, most of the variation (~ 70% or more; see also the spread of points in Figures S2b–S5b) could not be explained, despite the fact that many predictor variables were considered. We can only speculate about additional factors that may cause population divergence. One explanation may be that habitat conditions other than the ones included may cause strong divergent selection or limit dispersal between populations. Such conditions should be measured at much smaller scales than those used in our study and may include microhabitat characteristics such as the grain size of crops grown, types of cattle feed used, and available to this granivorous species, or food availability, which was found to be related to population divergence in a valley in France (Liu et al., 2013). A similar result was found for rural and urban populations in Hungary, but common garden experiments suggested that food availability did not result in a short‐term response in body mass (Liker, Papp, Bokony, & Lendvai, 2008). The high level of heterogeneity of the landscape mosaic in Romania and Bulgaria suggests that the process of local adaptation may occur at relatively small scales in those countries. If so, our estimate of the relative importance of IBA in population divergence is conservative. Another category of factors that may explain the remaining variation is related to chance events that are not linked to long‐term environmental conditions or the distance between populations, such as population demographic fluctuations or isolation by colonization (IBC; De Meester, Gómez, Okamura, & Schwenk, 2002; Orsini et al., 2013). Under IBC, a signal of founder effects can persist over time due to monopolization, where local adaptation is based only on standing genetic variation present in the first colonizers. However, relatively high population divergence is expected under such a scenario, which does not seem to be the case in our study region. Finally, morphological characteristics may rather be shaped by sexual than by environmental selection. Most studies on sexual selection in house sparrows have focused on the size of the black patch on males’ chests and on the white wing stripe, but females have also been shown to prefer larger males in some populations (Moreno‐Rueda & Hoi, 2012). Although the morphological traits measured here have not been implicated in sexual selection so far, it is conceivable that at least part of the divergence—in particular in the size component in males—can be attributed to differences in mate preferences between populations.

### 4.2 Influence of human habitation

Measures of human habitation appear to have little effect (positive or negative) on population divergence of house sparrows in Romania and Bulgaria. First, although road density was selected as a predictor in microsatellite variation and in the shape component PC2 in females, it did not contribute much to explaining the observed variation. Similarly, human population density was among the predictors in the model for microsatellite variation, but this variable retained a comparatively low importance score. Even though we have not sampled house sparrows in cities and thus lack information on this extreme end of the range of niches available along gradients in human‐dominated landscapes, our results are broadly concordant with those of Vangestel et al. (2012), who found no evidence for divergence between urban and rural house sparrow populations in Belgium (but see e.g., Liker et al., 2008 for morphological characteristics). Second, despite a lack of clear genetic structure, we expected that trends in genetic and morphological variation would be correlated to dispersal pathways facilitated by human habitation. For instance, Schrey, Liebl, Richards, and Martin (2014) found evidence that population expansion of house sparrows in Kenya could be explained by human‐mediated dispersal. However, in our study, resistance distances based on human habitation were not included in any of the models, suggesting that dispersal in these populations is not limited nor mediated by human activities.

### 4.3 Conservation recommendations

Although house sparrows are listed by the IUCN as of least concern (IUCN 2015), their populations are declining, most notably in their native range (Anderson, 2006; De Laet & Summers‐Smith, 2007; Murgui & Macias, 2010). The underlying causes of their decline remain poorly understood, but may be related to predation, competition, disease occurrence (Kruszewicz, Kruszewicz, Pawiak, & Mazurkiewicz, 1995), an increase in pollution (Summers‐Smith, 1999), and changes in anthropogenic activity that led to a shortage in food sources (Hole et al., 2002) and nest sites (Siriwardena, Robinson, & Crick, 2002). While house sparrows currently appear to be abundant in Romania and Bulgaria, the ongoing modernization of agriculture (Ioras, 2003) and predicted climate change may thus impact their numbers and require adaptive genetic or phenotypic changes. Moreover, intraspecific variation in these house sparrows may be a surrogate for that in other, less common species. In our preliminary and qualitative assessment, we found that environmentally associated intraspecific variation is likely insufficiently protected (Figure S6; Appendix S1). Particular conservation attention is warranted for lowland areas bordering the Danube River in the west, and the elevation gradient along the southern Carpathian Mountains. The results from the current study will be incorporated in much more detail in forthcoming work aiming at prioritizing areas for conservation in this biologically rich region, unique for Europe (e.g., Iojă et al., 2010; Wilson, Peet, Dengler, & Pärtel, 2012).

In summary, we found that selection by environmental variables, but not IBDL, is the main driver of population divergence in Romanian and Bulgarian house sparrow populations. Variables related to climate and vegetation best explained intraspecific variation, whereas those related to human habitation contributed comparatively little. Our study thus contributes to a growing body of literature suggesting that divergent selection may be a key driver of population divergence in many species and populations, and it improves our understanding of the spatial patterns and drivers of biodiversity in an understudied region.

## Conflict of Interest

None declared.

## Data Accessibility

Environmental variables , morphological data, and microsatellite genotypes: Dryad doi: 10.5061/dryad.9qk7d.
