# Supplementary figures and images for: Environmental selection is a main driver of divergence in house sparrows (Passer domesticus) in Romania and Bulgaria
Source: Ecol Evol. 2016 Oct 11;6(22):7954–64. doi: 10.1002/ece3.2509 (PMC5108248; doi:10.1002/ece3.2509)

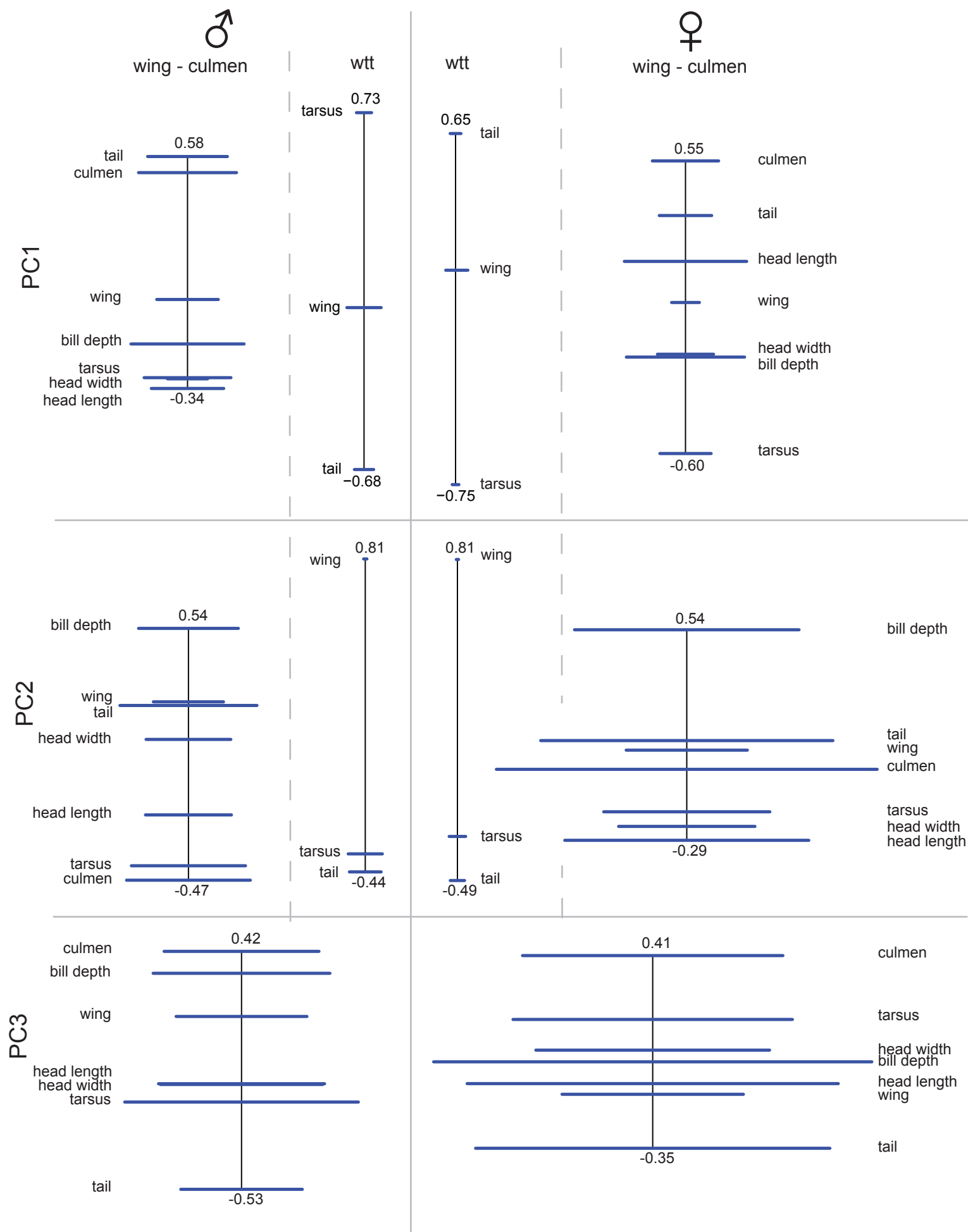

Fig. S1

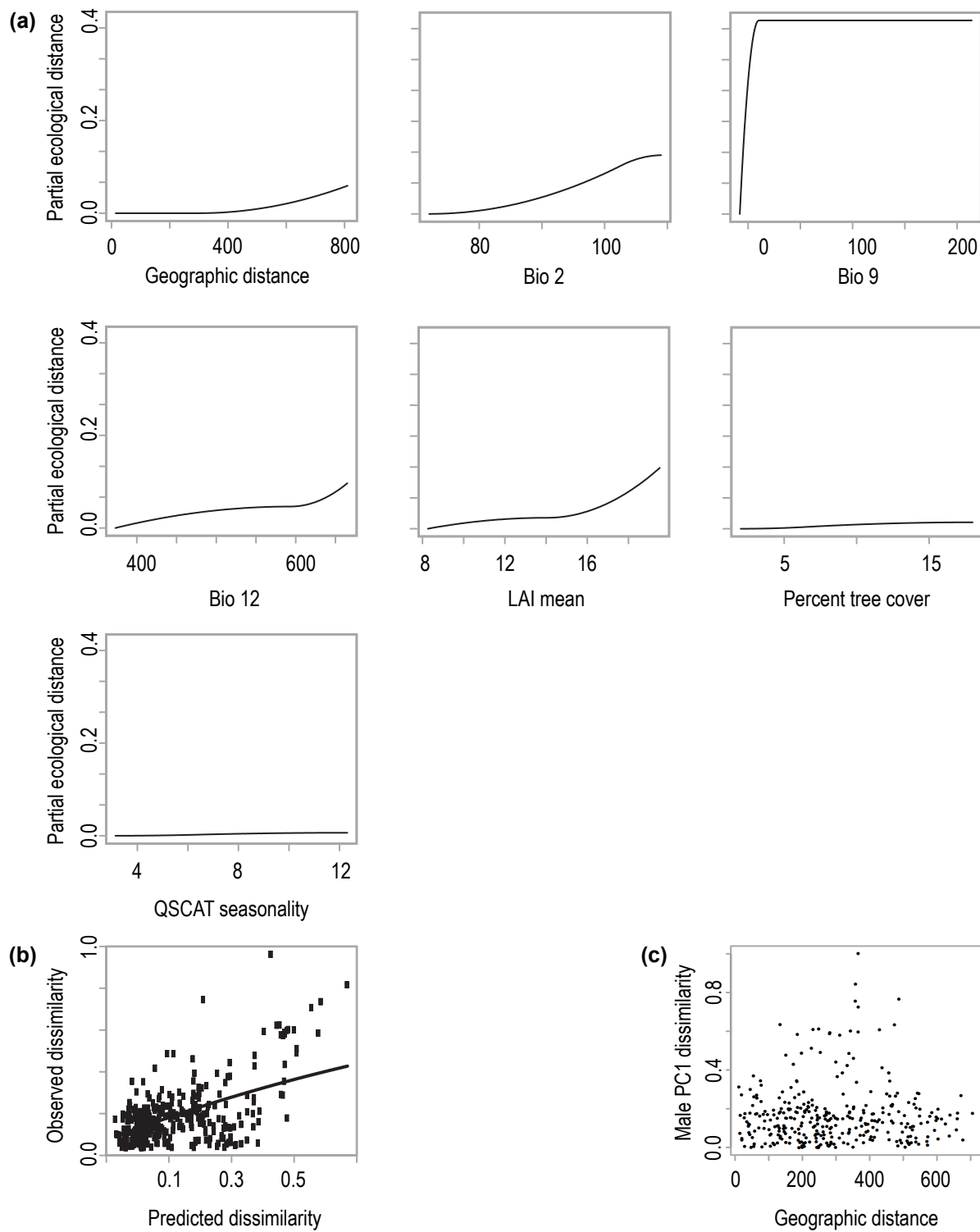

Fig. S2

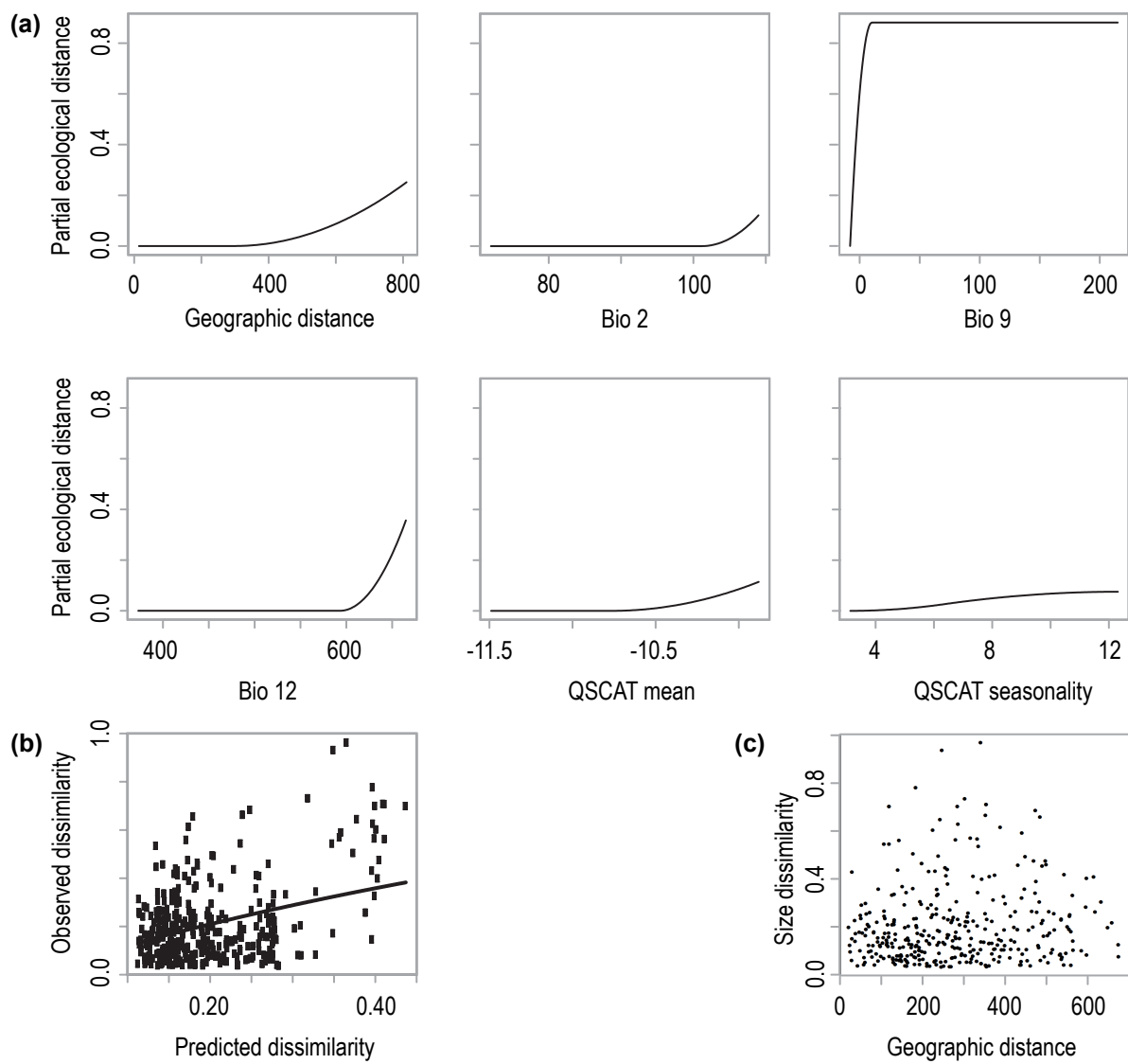

Fig. S3

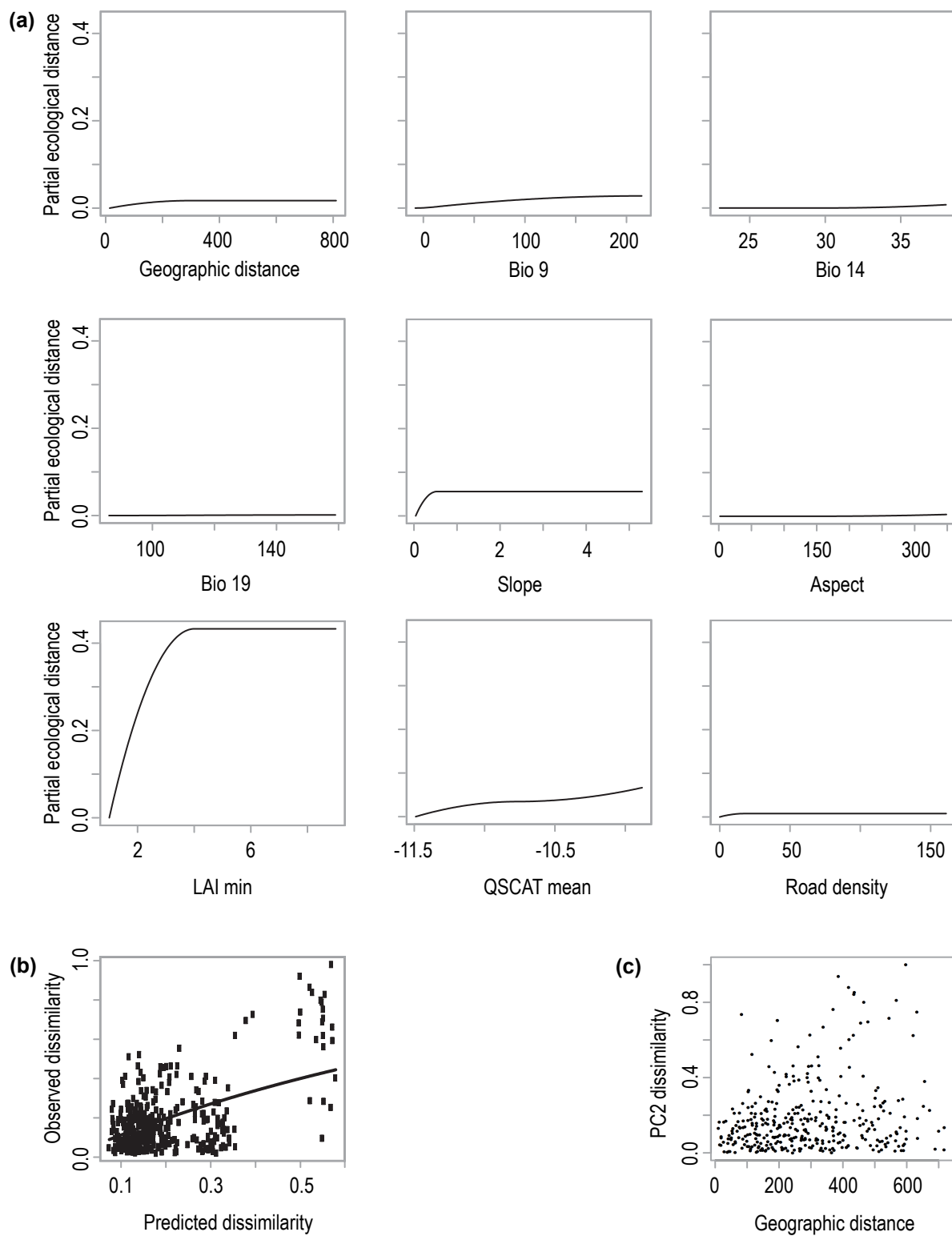

Fig. S4

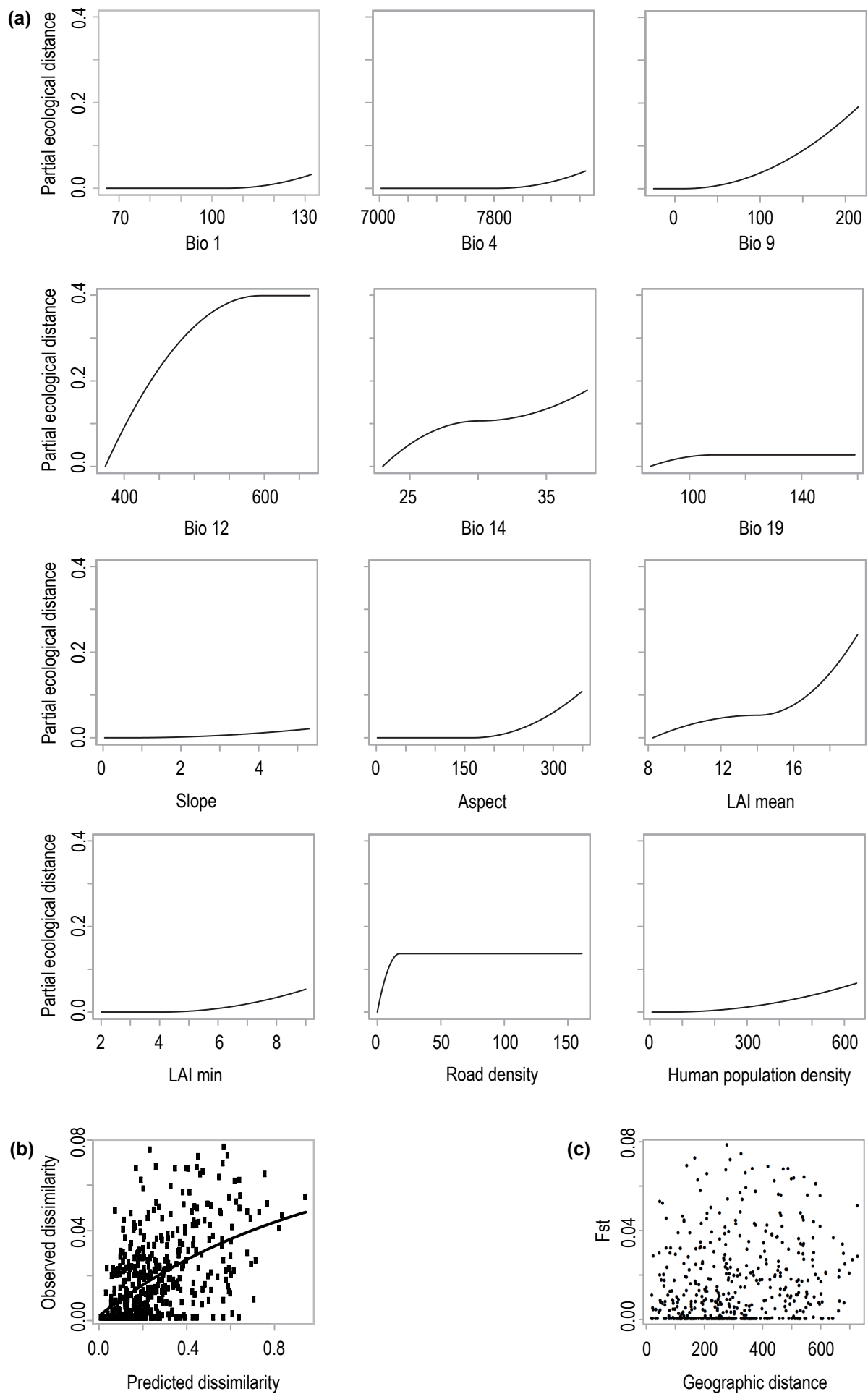

Fig. S5

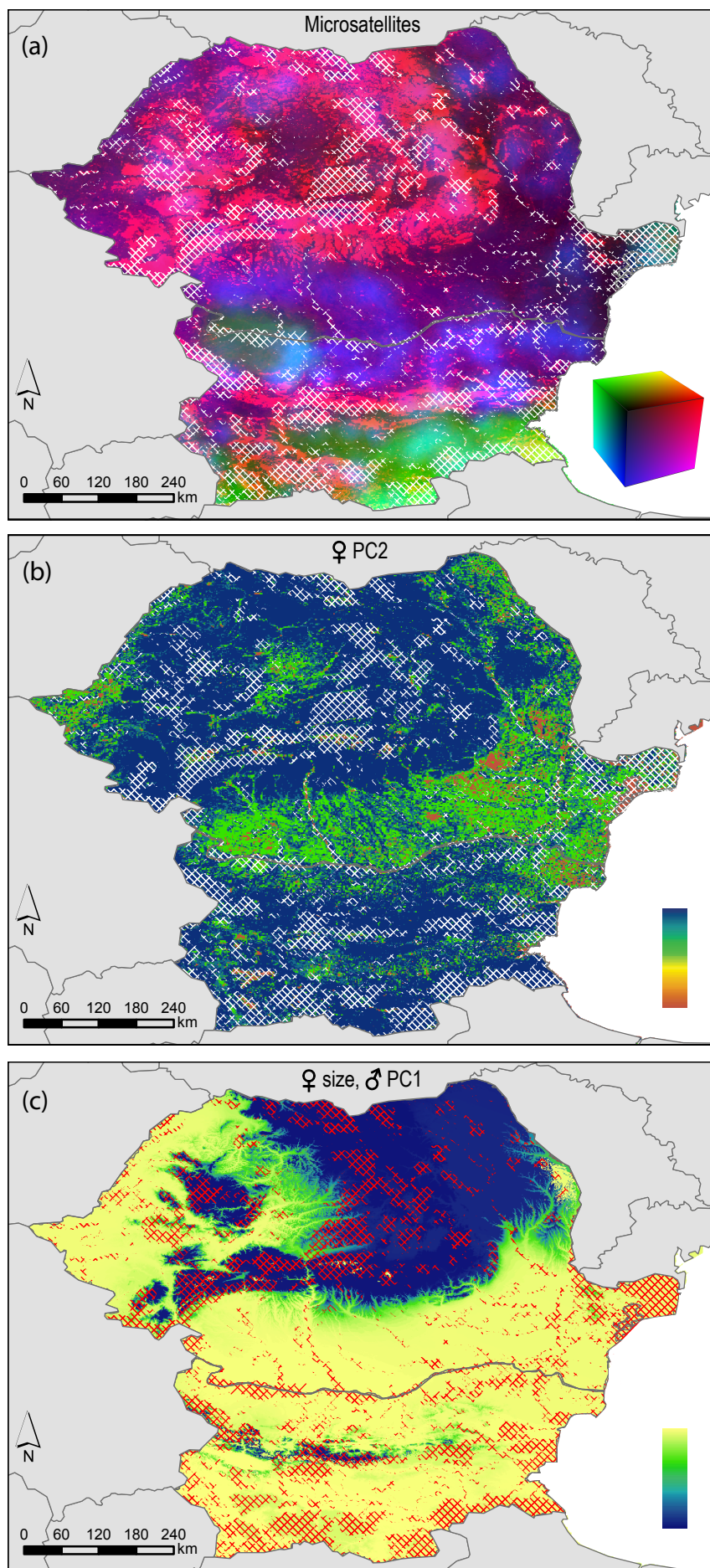

Fig. S6

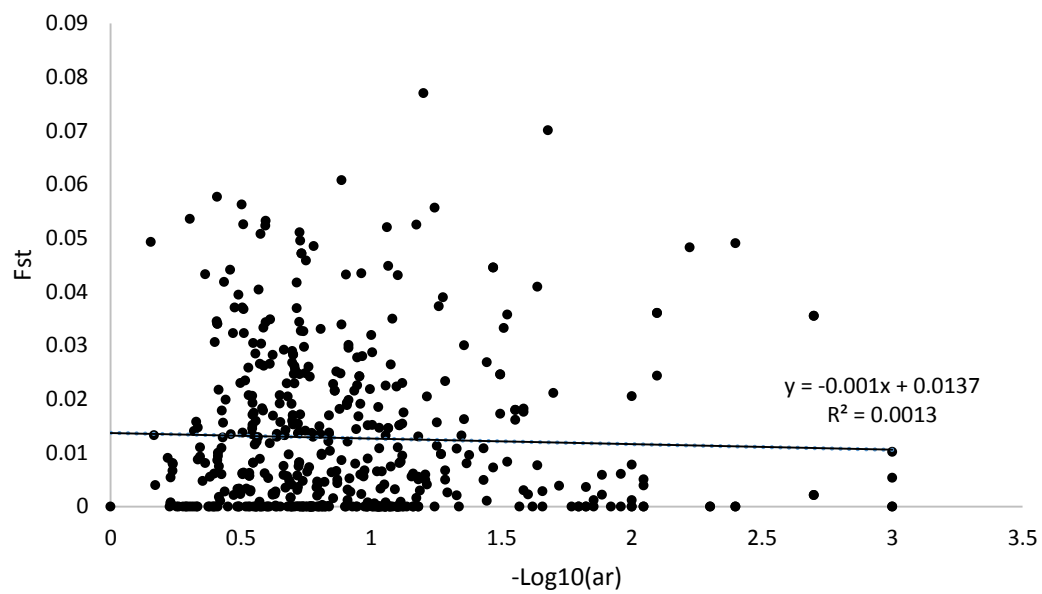

Fig. S7

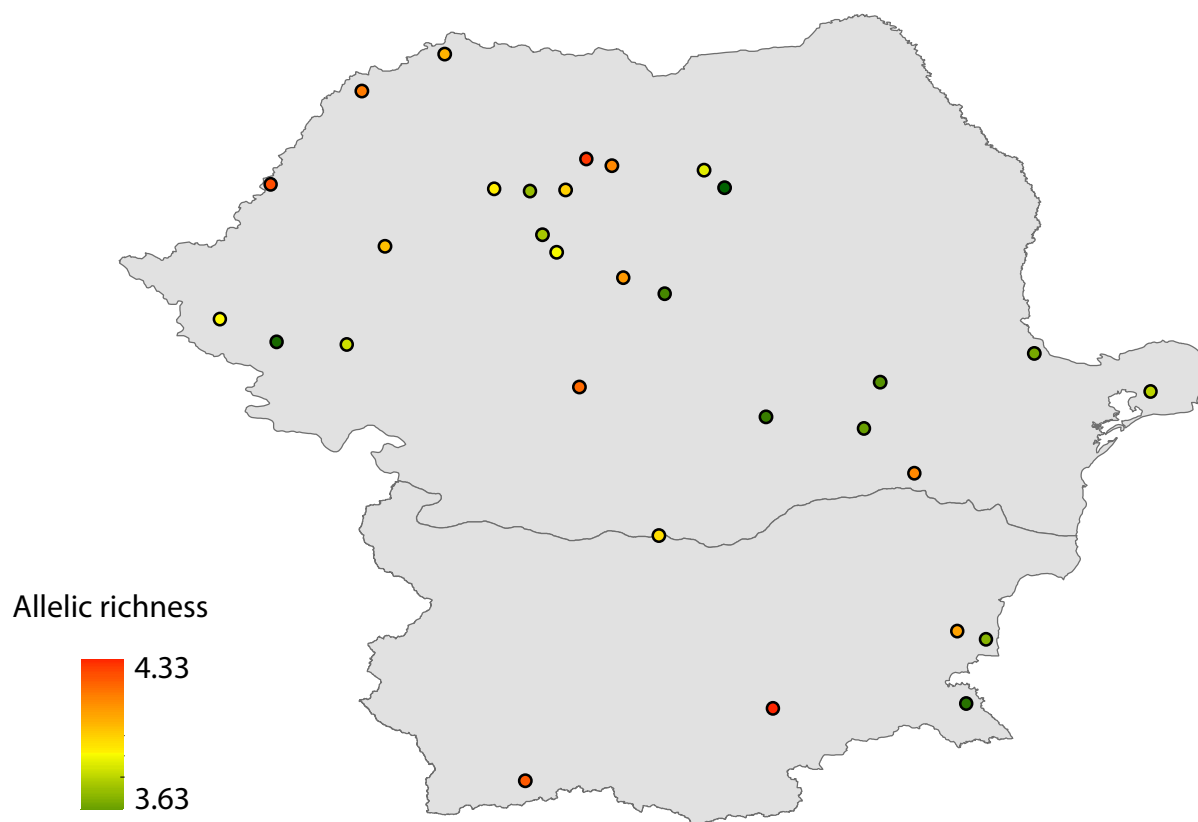

Fig. S8

Supplement: Supplementary file 1 [file ECE3-6-7954-s001.pdf]
