# Supplementary material for: Environmental selection is a main driver of divergence in house sparrows (Passer domesticus) in Romania and Bulgaria
Source: Ecol Evol. 2016 Oct 11;6(22):7954–64. doi: 10.1002/ece3.2509 (PMC5108248; doi:10.1002/ece3.2509)
Supplement: Supplementary file 2 [file ECE3-6-7954-s002.doc]

**Supporting information: Tables**

Table S1 Overview of sampling locations including coordinates and number of sampled individuals. Locations 1 to 5 are situated in Bulgaria and locations 6 to 33 in Romania. Locations 24 and 25, as well as 29 and 30 (bold) were pooled together because of small sampling size and/or they were close together.

|  |  |  | | No. of individuals | | | | |
| --- | --- | --- | --- | --- | --- | --- | --- | --- |
|  | Location | Latitude | Longitude | Total | Males | | Females | Unknown |
| 1 | Ognyanovo | 41.61487 | 23.79100 | 7 | 7 | | 0 | 0 |
| 2 | Beli bryag | 42.24340 | 25.94131 | 3 | 3 | | 0 | 0 |
| 3 | Jasna poljana | 42.28058 | 27.61904 | 6 | 0 | | 6 | 0 |
| 4 | Popovits | 42.83530 | 27.78912 | 8 | 1 | | 2 | 5 |
| 5 | Golica | 42.91410 | 27.54206 | 16 | 16 | | 0 | 0 |
| 6 | Poiana | 43.73681 | 24.95477 | 3 | 3 | | 0 | 0 |
| 7 | Berzovia | 45.42264 | 21.62602 | 3 | 3 | | 0 | 0 |
| 8 | Cluj Napoca | 46.74912 | 23.52103 | 31 | 15 | | 15 | 1 |
| 9 | Cojocna | 46.73354 | 23.82930 | 33 | 12 | | 20 | 1 |
| 10 | Vama Seăca | 46.35025 | 23.93556 | 40 | 15 | | 15 | 10 |
| 11 | Bălcaciu | 46.19968 | 24.05896 | 34 | 15 | | 15 | 4 |
| 12 | Agnita | 45.98115 | 24.64165 | 33 | 15 | | 15 | 3 |
| 13 | Făgăraș | 45.84098 | 25.00389 | 31 | 15 | | 15 | 1 |
| 14 | Independența | 44.28193 | 27.16513 | 30 | 15 | | 15 | 0 |
| 15 | Văcăreni | 45.31745 | 28.20522 | 40 | 15 | | 15 | 10 |
| 16 | Broșteni | 44.66979 | 26.73272 | 30 | 15 | | 15 | 0 |
| 17 | Cornești | 44.76584 | 25.88395 | 30 | 16 | | 14 | 0 |
| 18 | Balda | 46.73661 | 24.14184 | 29 | 15 | | 14 | 0 |
| 19 | Pinticu | 46.95053 | 24.54299 | 33 | 17 | | 16 | 0 |
| 20 | Lechința | 47.01407 | 24.32037 | 30 | 15 | | 15 | 0 |
| 21 | Turulung | 47.92441 | 23.08823 | 32 | 16 | | 16 | 0 |
| 22 | Petrești | 47.59987 | 22.37137 | 31 | 15 | | 16 | 0 |
| 23 | Salonta | 46.78522 | 21.58472 | 25 | 11 | | 10 | 4 |
| **24** | **Parța1** | **45.61900** | **21.14103** | **11** | **1** | | **9** | **1** |
| **25** | **Parța2** | **45.64539** | **21.13206** | **19** | **4** | | **7** | **8** |
| 26 | Caransebeș | 45.40022 | 22.24125 | 28 | 14 | | 10 | 4 |
| 27 | Mihăești | 45.02586 | 24.25767 | 31 | 18 | | 13 | 0 |
| 28 | Hălmagiu | 46.25341 | 22.56737 | 14 | 0 | | 6 | 8 |
| **29** | **Măgheruș** | **46.91388** | **25.33898** | **3** | **1** | | **2** | **0** |
| **30** | **Runc** | **46.89917** | **25.44515** | **2** | **0** | | **2** | **0** |
| 31 | Lăzarea | 46.75690 | 25.52250 | 3 | 2 | | 1 | 0 |
| 32 | Tintești | 45.06982 | 26.86985 | 15 | 0 | | 0 | 15 |
| 33 | Dunavățu de Jos | 44.98804 | 29.21840 | 7 | 4 | | 3 | 0 |
|  | total |  | | 691 | | 314 | 302 | 75 |

**Table S2** Microsatellite loci used, with repeat unit (motif), primers, range of allele sizes, corresponding annealing temperatures, references, and the multiplex primer mix it was used it. Loci highlighted in bold were used in the final analyses.

| Primer | Motif | Forward / reverse primers | Allele size (bp) | PCR-Temp | Reference | Primer combination |
| --- | --- | --- | --- | --- | --- | --- |
| Pdo31 | CTCA | GATCCACAGACGCAGACACAG  CATGCTGAATACTTTGTGAACTTGC | 232-279 | 55°C | Dawson et al. 2012 | PM 1 |
| **Pdo75** | **GAAA** | **GCATGACCTACAAACAGTTGC**  **TCCACCTATCTGATTCTGTCAAG** | **94-149** | **55°C** | **Dawson et al. 2012** | **PM 1** |
| **Pdoμ3** | **CCAT** | **CTGTTCATTAACTCACAGGT**  **AGTGAAACTTTAATCAGTTG** | **140** | **55°C** | **Griffith et al. 1999** | **PM 4** |
| **PdoA06** | **GT** | **GGCTTGAAGGACAGTGTATG**  **TTTCAAAAGGCACAGGTCT** | **103** | **56°C** | **Garnier et al. 2009** | **PM 3** |
| **PdoA08** | **TG** | **AGCTTTTCAGGTCTCCTTCT**  **CTACACCAGCAAGATCCATT** | **189** | **56°C** | **Garnier et al. 2009** | **PM 3** |
| PdoH05 | AC | CAAAGAATTTAAGGGGTGAA  ATGAACAACTCTCCAGCATC | 151 | 56°C | Garnier et al. 2009 | PM 3 |
| Pdo7 | TTTC | AAATGCAAATAAATGTGCGG  GGCAAAGCCTTCCTTATCTC | 162-285 | 60°C | Griffith et al. 2007 | PM 5 |
| **Pdo10** | **CA** | **AATGTGAATCCCTCCAGAAAC**  **ATGGAGTTTGGGGAATGG** | **113-147** | **60°C** | **Griffith et al. 2007** | **PM 6** |
| **Pdo46** | **CA** | **GTGGGTGTGCCTGAAGATGTG**  **AGCGGGTCAGGAGCCTCTC** | **191-211** | **60°C** | **Dawson et al. 2012** | **PM 6** |
| **Pdo16** | **CA** | **GTGTATATGCAAATGACAAGACCAAAGC**  **TCACGCTGACCTAGATGCTATCAGAG** | **282-297** | **60°C** | **Dawson et al. 2012** | **PM 2** |
| **Pdo36** | **GT** | **GCATTCAAAAATGGCAAGAGGA**  **GAGGCTACCCCTTTCCTGAACA** | **183-221** | **60°C** | **Dawson et al. 2012** | **PM 2** |
| PdoF05 | TG | GCATATTTCTGGCATTCTTC  TCAAATAAAGTGCTCCACAA | 103 | 60°C | Garnier et al. 2009 | PM 2 |

**Table S3** Environmental variables obtained for this study. Variables highlighted in bold were included in generalized dissimilarity models, after highly cross-correlated ones (with Pearson correlation coefficients > 0.7) were omitted.

| Name | Attribute | Source |
| --- | --- | --- |
| **Bio 1** | **Annual mean temperature** | **WorldClim** |
| **Bio 2** | **Mean diurnal range [mean of monthly (max temp – min temp)]** | **WorldClim** |
| Bio 3 | Isothermality [(Bio2/Bio7) * 100] | WorldClim |
| **Bio 4** | **Temperature seasonality (standard deviation*100)** | **WorldClim** |
| Bio 5 | Maximum temperature of the warmest month | WorldClim |
| Bio 6 | Minimum temperature of the coldest month | WorldClim |
| Bio 7 | Temperature annual range (Bio5-Bio6) | WorldClim |
| Bio 8 | Mean temperature of the wettest quarter | WorldClim |
| **Bio 9** | **Mean temperature of the driest quarter** | **WorldClim** |
| Bio 10 | Mean temperature of the warmest quarter | WorldClim |
| Bio 11 | Mean temperature of the coldest quarter | WorldClim |
| **Bio 12** | **Annual precipitation** | **WorldClim** |
| Bio 13 | Precipitation of the wettest month | WorldClim |
| **Bio 14** | **Precipitation of the driest month** | **WorldClim** |
| Bio 15 | Precipitation seasonality (coefficient of variation) | WorldClim |
| Bio 16 | Precipitation of the wettest quarter | WorldClim |
| Bio 17 | Precipitation of the driest quarter | WorldClim |
| Bio 18 | Precipitation of the warmest quarter | WorldClim |
| **Bio 19** | **Precipitation of the coldest quarter** | **WorldClim** |
| ELV | Elevation | SRTM |
| **SLOPE** | **Slope** | **SRTM** |
| **ASPECT** | **Aspect** | **SRTM** |
| **TREE2001** | **Percent tree cover** | **Global Land Cover facility** |
| LAI sd | Lead Area Index standard deviation | Global Land Cover facility |
| **LAI min** | **Leaf Area Index minimum** | **Global Land Cover facility** |
| **LAI mean** | **Leaf Area Index mean** | **Global Land Cover facility** |
| LAI max | Leaf Area Index maximum | Global Land Cover facility |
| QSCAT min | Quickscat minimum | NASA SCP |
| **QSCAT mean** | Quickscat mean | NASA SCP |
| **QSCAT max** | Quickscat maximum | NASA SCP |
| **QSCAT seasonality** | Quickscat seasonality (coefficient of variation) | NASA SCP |
| **Road density** | **Road density** | Digital Chart of the World |
| **Danube barrier** | **Danube as barrier** |  |
| **Human pop dens** | **Human population density** | Gridded Population of the World |

Table S5 PCA results for the shape component of wing, tail, and tarsus lengths in males and females based on the three-variables-all-locations dataset. Numbers indicate factor loadings and proportion of variance explained. The first two axes explained all shape variation.

|  |  | Shape PC1 | Shape PC2 |
| --- | --- | --- | --- |
| Females | Wing length | 0.1049 | -0.8097 |
|  | Tail length | 0.6488 | 0.4957 |
|  | Tarsus length | -0.7537 | 0.3140 |
|  | Prop Var | 0.8142 | 0.1858 |
| Males | Wing length | -0.0396 | 0.8155 |
|  | Tail length | -0.6865 | -0.4421 |
|  | Tarsus length | 0.7261 | -0.3734 |
|  | Prop Var | 0.7261 | 0.2739 |

Prop Var = Proportion of variance explained by each morphological shape component.

Table S6 Basic population genetic statistics for each sampling location. *N*_E_ = number of effective alleles; *H*_O_ = observed heterozygosity; *H*_E_ = expected heterozygosity; *F* = fixation index.

|  |  |  |  |  |  |
| --- | --- | --- | --- | --- | --- |
| Population |  | *N*_E_ | *H*_O_ | *H*_E_ | *F* |
| Agnita | Mean | 6.066 | 0.785 | 0.759 | -0.044 |
|  | SE | 0.857 | 0.087 | 0.085 | 0.034 |
| Bălcaciu | Mean | 5.094 | 0.846 | 0.771 | -0.094 |
|  | SE | 0.563 | 0.063 | 0.044 | 0.044 |
| Balda | Mean | 5.574 | 0.808 | 0.753 | -0.075 |
|  | SE | 0.713 | 0.093 | 0.082 | 0.042 |
| Beli bryag | Mean | 4.049 | 0.844 | 0.722 | -0.164 |
|  | SE | 0.482 | 0.066 | 0.040 | 0.056 |
| Berzovia | Mean | 3.220 | 0.708 | 0.639 | -0.099 |
|  | SE | 0.401 | 0.098 | 0.061 | 0.093 |
| Broșteni | Mean | 4.893 | 0.750 | 0.740 | 0.008 |
|  | SE | 0.708 | 0.087 | 0.062 | 0.084 |
| Caransebeș | Mean | 4.890 | 0.761 | 0.727 | -0.053 |
|  | SE | 0.728 | 0.087 | 0.078 | 0.042 |
| Cluj Napoca | Mean | 5.479 | 0.808 | 0.766 | -0.067 |
|  | SE | 0.701 | 0.061 | 0.063 | 0.034 |
| Cojocna | Mean | 5.291 | 0.757 | 0.725 | -0.043 |
|  | SE | 0.649 | 0.117 | 0.104 | 0.055 |
| Cornești | Mean | 5.618 | 0.725 | 0.726 | 0.000 |
|  | SE | 0.834 | 0.108 | 0.105 | 0.039 |
| Dunavățu de Jos | Mean | 4.558 | 0.625 | 0.737 | 0.207 |
|  | SE | 0.581 | 0.117 | 0.052 | 0.142 |
| Făgăraș | Mean | 5.124 | 0.698 | 0.732 | 0.028 |
|  | SE | 0.772 | 0.089 | 0.081 | 0.072 |
| Golica | Mean | 6.887 | 0.801 | 0.794 | 0.000 |
|  | SE | 0.928 | 0.079 | 0.070 | 0.033 |
| Hălmagiu | Mean | 6.477 | 0.731 | 0.767 | 0.034 |
|  | SE | 0.884 | 0.090 | 0.091 | 0.044 |

**Table S6** (continued)

| Independența | Mean | 5.988 | 0.740 | 0.777 | 0.030 |
| --- | --- | --- | --- | --- | --- |
|  | SE | 0.968 | 0.055 | 0.061 | 0.047 |
| Jasna poljana | Mean | 4.765 | 0.727 | 0.739 | 0.046 |
|  | SE | 0.697 | 0.084 | 0.054 | 0.062 |
| Lăzarea | Mean | 3.184 | 0.583 | 0.590 | 0.071 |
|  | SE | 0.550 | 0.137 | 0.094 | 0.151 |
| Lechința | Mean | 5.811 | 0.811 | 0.789 | -0.009 |
|  | SE | 0.630 | 0.076 | 0.054 | 0.048 |
| Măgheruș-Runc | Mean | 4.475 | 0.825 | 0.725 | -0.134 |
|  | SE | 0.587 | 0.096 | 0.061 | 0.080 |
| Mihăești | Mean | 5.552 | 0.750 | 0.758 | 0.041 |
|  | SE | 0.804 | 0.091 | 0.066 | 0.063 |
| Ognyanovo | Mean | 5.904 | 0.724 | 0.761 | 0.098 |
|  | SE | 1.001 | 0.106 | 0.067 | 0.092 |
| Parța | Mean | 5.332 | 0.788 | 0.768 | -0.039 |
|  | SE | 0.722 | 0.055 | 0.055 | 0.044 |
| Petrești | Mean | 5.788 | 0.829 | 0.770 | -0.082 |
|  | SE | 0.795 | 0.078 | 0.069 | 0.038 |
| Pinticu | Mean | 5.915 | 0.706 | 0.778 | 0.111 |
|  | SE | 0.929 | 0.072 | 0.056 | 0.052 |
| Poiana | Mean | 3.650 | 0.708 | 0.646 | -0.094 |
|  | SE | 0.515 | 0.133 | 0.094 | 0.120 |
| Popovits | Mean | 4.853 | 0.750 | 0.723 | -0.043 |
|  | SE | 0.749 | 0.075 | 0.070 | 0.035 |
| Salonta | Mean | 6.233 | 0.763 | 0.793 | 0.070 |
|  | SE | 0.881 | 0.091 | 0.055 | 0.078 |
| Tintești | Mean | 5.557 | 0.750 | 0.728 | -0.033 |
|  | SE | 0.883 | 0.096 | 0.092 | 0.021 |
| Turulung | Mean | 5.463 | 0.738 | 0.784 | 0.093 |
|  | SE | 0.663 | 0.096 | 0.040 | 0.107 |
| Văcăreni | Mean | 4.898 | 0.676 | 0.764 | 0.143 |
|  | SE | 0.641 | 0.102 | 0.037 | 0.118 |
| Vama Seacă | Mean | 5.278 | 0.813 | 0.733 | -0.102 |
|  | SE | 0.697 | 0.106 | 0.092 | 0.033 |
