# Supplementary material for: Environmental selection is a main driver of divergence in house sparrows (Passer domesticus) in Romania and Bulgaria
Source: Ecol Evol. 2016 Oct 11;6(22):7954–64. doi: 10.1002/ece3.2509 (PMC5108248; doi:10.1002/ece3.2509)
Supplement: Supplementary file 4 [file ECE3-6-7954-s004.docx]

**Appendix 1 - Supporting Materials and Methods**

*Genetic identification of* Passer domesticus *and* P. hispaniolensis

In the southern part of its range in Bulgaria, house sparrows (*Passer domesticus*) co-occur with the Spanish sparrow, *P. hispaniolensis*, and are even found breeding in mixed colonies. Males of the two species are easily distinguishable, but females are very similar in appearance. We, therefore, identified the species of females sampled in southern populations using molecular methods. To do so, we sequenced a 749-base pair region of the cytochrome c oxidase subunit I (COI) mitochondrial gene using the primers BirdF1 and BirdR1 ([Hebert *et al.* 2004](#_ENREF_9)). The COI gene is known for its relatively fast mutation rate and its use across many taxa for barcoding purposes.

The PCR reaction mix consisted of 2.5 µl 10× PCR buffer, 13.4 µl HPLC water, 1.0 µl dNTP’s (10 mM), 2.0 µl MgCl_2_ (25 mM), 1.0 µl BSA (20 mg/ml), 1.0 µl of each primer (0.1 mM), 0.1 µl Taq DNA polymerase and 3.0 µl extracted DNA per reaction. PCR conditions were: 94°C initial denaturation for 5 minutes; 39 cycles of 94°C denaturation for 40 seconds, 50°C annealing for 40 seconds, and 72°C extension for 1 minute; and 72°C final extension for 10 minutes.

PCR products were visualized using agarose gel electrophoresis to check for the amplification of the fragment. Successfully amplified PCR products were cleaned up using the Promega Wizard^©^ SV Gel and PCR Clean-Up System according to the manufacturer’s protocol. Cleaned up samples were then sent to LGC Genomics (Berlin, Germany) for sequencing. Resulting sequences with corresponding chromatograms were visualized and edited where necessary with Unipro UGENE v1.12.2 ([Okonechnikov *et al.* 2012](#_ENREF_12)). We used ClustalX 2.1 ([Thompson *et al.* 1997](#_ENREF_16); [Larkin *et al.* 2007](#_ENREF_10)) to align sequences with each other and with reference sequences for *P. domesticus*, *P. hispaniolensis*, and *P. montanus*, obtained from GenBank. We then used Mega v. 6 ([Tamura *et al.* 2013](#_ENREF_15)) to construct a Maximum Likelihood tree using the Hasegawa-Kishino-Yano (HKY) substitution model with uniform rates and 1000 bootstrap replicates. Individuals that were included in a monophyletic group with reference sequences of *P. domesticus* were considered members of the target species and included in further microsatellite analyses.

*Morphological measurements and analyses*

Morphological measures are unlikely to be completely independent from one another. If an individual grows isometrically, i.e. without changing shape, all measures will be affected equally. For multivariate analyses, it is therefore essential to distinguish between the isometric size component and the allometric (shape) component of these measures. Several methods are available to do so, such as Procrustes analyses on morphological landmarks, or standardization using an independent measure of body size. However, neither of these were available based on our collection methods, so we used the “PCA ratio spectrum” method developed by Baur & Leuenberger ([2011](#_ENREF_1)). This method identifies common patterns ‒ that could represent isometric growth ‒ among all possible ratios of morphological measurements, and returns independent PCA scores for one size and several shape components. For the size component and each shape component that explained > 10% of the total variation in the PCA, we computed population pairwise differences as follows: $\frac{\left| \bar{x}-\bar{y} \right|}{\sigma_{\bar{x}}+\sigma_{\bar{y}}}$ where $\bar{x}$ and $\bar{y}$ are the averages for populations $x$ and $y$, and $\sigma_{\bar{x}}$ and $\sigma_{\bar{y}}$ are their standard deviations. Because we only had partial datasets ‒ one with three morphological variables (wing, tail, and tarsus lengths) for all locations, and one with all morphological variables (also including culmen and head lengths, head width, and bill depth) for only nine locations ‒ we needed to decide which of those datasets was most appropriate to use. To further explore these datasets, we in fact ran PCA ratio spectra and generalized dissimilarity models for both and compared the results (not shown). We found that the percent of total variation explained by our models was about twice as high for the all-variables-nine-location dataset as compared to the three-variables-all-locations dataset. To investigate whether this could potentially be explained by the difference in the number of locations, we reduced the three-variables-all-locations dataset to only comprise the nine locations of the other dataset, and ran GDMs with this reduced dataset. We found that this data reduction method almost doubled the percent of total variation explained in GDMs. Although we cannot rule out that this is a real pattern among those nine locations, we suspected that these results were an artifact of entering too few locations in the models, and we only present the results for the three-variables-all-locations dataset, except for the results from PCA ratio spectra.

*Microsatellite analysis*

The extracted DNA was used to determine intraspecific genetic variation by genotyping an initial set of twelve microsatellite loci (Pdo31, Pdo75, Pdoµ3, PdoA06, PdoA08, PdoH05, Pdo7, Pdo10, Pdo16, Pdo36, Pdo46, and PdoF05) by using the M13-hybrid primer process ([Schuelke 2000](#_ENREF_14); [Boutin-Ganache *et al.* 2001](#_ENREF_2)). This procedure uses three types of primers: first a hybrid primer, which consists of the forward primer with a tagged-on M13F sequence (16p: 5’-GTAAAACGACGGCCAG-3’) on the 5’end; second the corresponding reverse primer; and third a dye labeled M13F primer, which is complementary to the M13F sequence. The primer mix consisted of 4 µl of the reverse primer (100 µM), 8 µl forward-M13 hybrid primer (2.5 µM), 8 µl M13 dye labeled primer (2.5 µM) and 180 µl water. To run the multiplex PCR, a PCR reaction mix was made, for each 10 µl reaction consisting of 1.0 µl primer mix, 2.1 µl water, 0.4 µl Bovine Serum Albumin, and 5.0 µl Qiagen Multiplex Mastermix added to 1.5 µl sample DNA. The PCR was then run as a two-stage cycle starting with 15 minutes at 95°C, followed by the first cycle which consists of three steps: 30 seconds at 94°C, 90 seconds at 55/56/60°C (depending on the primer mix) and 60 seconds at 72°C, repeated 25 times. The second cycle also consisted of three steps and was conducted as follows: 94°C for 30 seconds, then 90 seconds at 53°C and finally 60 seconds at 72°C, repeated 20 times. After these cycles, a final step of 60°C for 30 minutes was run.

Fragment analysis was carried out on an ABI 3730 sequencer at the University of Turku, Finland. Results were analyzed with GeneMarker V2.4.1 (Softgenetics, State College, PA). We used the following peak detection settings: detection range between 100 and 400 base pairs (bp); peak detection threshold with an intensity between 100 and 8000; stutter peak filter of 5% on the left side and 40% on the right side. The detected peaks were then examined visually, and edited where necessary.

*Population genetic structure*

To assess the level of genetic structure, we conducted two Bayesian clustering analyses. First, we ran STRUCTURE ([Pritchard *et al.* 2000](#_ENREF_13)), which uses the genotypic data only and is capable of incorporating putative population origin as prior information, but does not incorporate the geographic sampling location of individuals. We ran five independent runs of 500,000 iterations after a burn-in of 50,000 iterations, exploring values of *K* - the assumed number of different genetic clusters - ranging from *K* = 1 to *K* = 31, which corresponds to the maximum number of sampling locations. In two different analyses, we assumed an admixture model with either correlated or uncorrelated allele frequencies and the sampling site as prior information (LOCPRIOR). The most likely number of clusters was determined using the method proposed by [Evanno *et al.* (2005](#_ENREF_4)) in STRUCTURE HARVESTER ([Earl & vonHoldt 2012](#_ENREF_3)).

The second Bayesian clustering method we implemented was GENELAND 4.0 ([Guillot *et al.* 2005a](#_ENREF_6); [Guillot *et al.* 2005b](#_ENREF_7); [Guillot *et al.* 2008](#_ENREF_8)), run in R 3.1.2 (R Core Team 2015). In contrast to STRUCTURE, GENELAND explicitly takes into account the spatial location and orientation of samples. Here, we also assumed an admixture model with either correlated or uncorrelated allele frequencies. Because MICRO-CHECKER results suggested the presence of null alleles, we also implemented a null allele model, which attempts to correct for the false identification of genetic structure as a result of the presence of an excess of homozygotes due to null alleles. We ran ten independent runs of 500,000 iterations, thinning of 100, and a burn-in of 50,000 for *K* = 1 - 31.

*Influence of the number of loci on estimates of F_ST_*

The genetic data used here consists of a relatively small set of eight microsatellite markers. To get insight into the robustness of this data set, we computed *F*_ST_ values of further reduced data sets consisting of all possible combinations of six and seven loci, and correlated these *F*_ST_ values with those from the full set of eight loci. The average R^2^ of correlations between all sets of seven loci and the full data set was 0.87 (SD = 0.11; median R^2^ = 0.93); the average R^2^ of correlations between all 28 sets of six loci and the full data set was 0.75 (SD = 0.14; median R^2^ = 0.76). These results suggest that a general trend of genetic divergence is detectable in our data set, and that this trend is conveyed by different combinations of microsatellite loci. We may thus expect that this same trend continues to be found when more loci are added, and therefore that our results of subsequent landscape genetic analyses are relatively robust, even with the data set presented here.

*F_st_ versus allelic richness*

The level of divergence between populations is susceptible to differences in alpha diversity (e.g. allelic richness) within populations. Lower alpha diversity may increase divergence, in particular when divergence is a result from genetic drift after for instance a bottleneck event. Such a scenario would result in spurious correlations between *F*_st_ and environmental variables if bottleneck or founder events systematically occur in a certain type of environment. To assess whether GDM correlations between *F*_st_ values and environmental variables could be attributed to differences in alpha diversity within populations, we used the following two approaches.

1. We calculated overall allelic richness (ar) across all loci per sampling site. We then calculated location-pairwise differences in ar and plotted the negative log-transformed values against the corresponding *F*_st_ values (Fig. S7). If alpha diversity is affecting beta diversity, we would expect a positive correlation between ar and *F*_st_. However, we did not find a significant correlation between these variables (slope = -0.001, R^2^ = 0.0013).
2. Even given the results above, it is conceivable that populations in different environments exhibit different levels of allelic richness. We, therefore, also tested for linear correlations between ar and environmental variables using the “lm” function in R, and found no significant linear model (P > 0.05 for each variable; overall adjusted R^2^ = -0.117, F = 0.8255, P = 0.6537).

From these results we concluded that correlations between F_st_ and environmental heterogeneity were not the result of differences in population-wise genetic variation (i.e. alpha diversity; allelic richness).

*Range expansion*

Demographic processes may influence genetic diversity within and between populations, and as a result could confound tests for selection and IBD. Since the Last Glacial Maximum, house sparrows have likely expanded their range from southern refugia, or even from a refugium within the Carpathian Mountains. Range expansions are expected to result in lowered genetic variation in peripheral populations, in the direction of the expansion. Even though it appeared unlikely that a signal of past range expansion would still be present in these southern European populations after thousands of generations, we visually assessed the geographic pattern of allelic richness (which was similar between populations, ranging from 3.63 – 4.33) by plotting ar per population on a map (Fig. S8). If a signal of range expansion would still be present, we would have expected to see diminishing ar from south to north or away from the Carpathian Mountains. However, we found no such pattern, and concluded that past range expansion and associated effects on intraspecific variation is unlikely to affect our landscape genetic analyses.

*Environmental variables - computation of QSCAT*

A measure of surface moisture and canopy roughness (over dense forest) was obtained from the QuikScat microwave instrument (QSCAT; ([Long *et al.* 2001](#_ENREF_11))). We computed multi-year (2000 ‒ 2008) averages of raw backscatter measurements at the horizontal polarization. To do so, daily data records for Europe for the years 2000 ‒ 2008 were downloaded from the BYU Scatterometer Climate Record Pathfinder database (<http://www.scp.byu.edu/data/Quikscat/SIRv2/qush/Eur.html>). The downloaded .SIR datafiles were converted to GeoTIFF files using “sir_utils”, available from NASA SCP (<http://www.scp.byu.edu/docs/geotiff.html>). Further processing was then done in ArcGIS 10.2.2 (ESRI, Redlands, USA). We visually inspected daily images for potential anomalies, such as large areas with missing data and geometric patterns that could indicate an error of the sensor. Out of the 365 daily images, a minimum of 0 and maximum of 7 images showed errors and were omitted from further processing. We then computed yearly averages, minima, maxima, and seasonality for each year; we subsequently averaged these variables across the years 2000 ‒ 2008. To focus more on climatology and less on short-term extreme weather events, we did not use the daily images to compute minima and maxima, but first calculate two-week averages, of which we then took the minima and maxima. Finally, to compute QSCAT seasonality, we used the coefficient of variation, analogous to the WorldClim variable Bio 15: precipitation seasonality (www.worldclim.org/bioclim). We first computed monthly averages and then defined seasonality as: 100 × (SD_monthly mean_ / |average of monthly mean|).

*Visualization of biotic variation across the landscape*

To visualize biotic variation across our study area, we used the approach from [Fitzpatrick & Keller (2015](#_ENREF_5)). In brief, the turnover functions derived from GDMs were used to transform the retained environmental variables into indices of genetic importance. These genetic importance values were subsequently reduced into orthogonal axes by a Principal Components Analysis (PCA), and the first three axes were mapped to the red, green, and blue channels in a RGB composite layer. One caveat of this approach is that not all variation in the genetic importance variables is explained by the first three PC axes.

A more proper way to visualize biotic variation is to use the ‘predict.gdm’ function in the ‘gdm’ package with subsequent multidimensional scaling to reduce the n-dimensional GDM matrix into three dimensions that can be mapped to RGB values. However, in the current set-up (R package ‘gdm’ version 1.1.2, run on a stand-alone PC with 8 Gb of RAM), it was only possible to do so for a maximum of 3000 locations. To evaluate whether the high resolution spatial projections from the approach from ([Fitzpatrick & Keller 2015](#_ENREF_5)) were broadly consistent with those at low resolution using the ‘predict.gdm’ function, we created maps using both approaches and visually compared the results. We extracted the values of environmental variables at 3000 locations randomly distributed across the study area. We then predicted the biotic response between those 3000 locations based on the GDM turnover functions, and subsequently used the ‘cmdscale’ function in R to reduce the 3000-dimensional GDM matrix into three dimensions. We created spatial interpolations for each of these dimensions using Empirical Bayesian Kriging (EBK) in ArcGIS 10.2.2 (ESRI, Redlands, USA). EBK accounts for the error in estimating the semivariogram model by estimating and using many different semivariograms and attempting to find the optimal parameters for the spatial interpolation. We used EBK with no transformation; a power semivariogram; maximum number of points in each local model: 100; local model area overlap factor: 1; number of simulated semivariograms: 100; standard circular search neighborhood; maximum 15 neighbors. Resulting maps were visually very similar to those obtained through ordinary kriging with a spherical semivariogram, variable search radius, and twelve points in the search radius. Maps for each of the three dimensions were combined into a composite RGB map.

The spatial projections of biotic variation were highly consistent between methods (results not shown). As a result, we only show the high resolution maps obtained through a transformation of environmental variables into genetic importance values and a subsequent PCA.

*Conservation of intraspecific variation*

We visually compared the overlap of protected areas in Romania and Bulgaria with the full range of environmentally associated intraspecific variation in house sparrows. This evaluation is by no means meant to be a detailed analysis of the protection status of intraspecific variation, but can serve as a preliminary and qualitative assessment. The current study will contribute to a larger forthcoming comparative study of multiple species, integrating intraspecific variation with other measures of biodiversity.

We plotted protected areas recorded in the Natura 2000 database (<http://natura2000.eea.europa.eu/>), updated till 2014, on the maps of GDM results (Fig. S6). We did not distinguish between the protection status of sites, or whether protection had been implemented. For both genetic and morphological variation incomplete coverage of the full range of variation (all colors in the maps) can be observed. Insufficient protection of genetic variation and variation in morphology in females (PC2) is suggested in the lowland areas bordering the Danube River, where protected areas only to a small extent overlap with the blue-black colors (microsatellites; Fig. S6a) and the green and brown colors (female PC2; Fig. S6b). In addition, female size and the first shape component in males change rapidly along the elevation gradient in the southern Carpathian Mountains, but hardly any of these areas are under protection (Fig. S6c).

**References**

Baur H, Leuenberger C (2011) Analysis of ratios in multivariate morphometry. *Systematic Biology* **60**, 813-825.

Boutin-Ganache I, Raposo M, Raymond M, Deschepper CF (2001) M13-tailed primers improve the readability and usability of microsatellite analyses performed with two different allele-sizing methods. *BioTechniques* **31**, 24-26, 28.

Earl D, vonHoldt B (2012) STRUCTURE HARVESTER: a website and program for visualizing STRUCTURE output and implementing the Evanno method. *Conservation Genetics Resources* **4**, 359-361.

Evanno G, Regnaut S, Goudet J (2005) Detecting the number of clusters of individuals using the software STRUCTURE: a simulation study. *Molecular Ecology* **14**, 2611-2620.

Fitzpatrick MC, Keller SR (2015) Ecological genomics meets community-level modelling of biodiversity: mapping the genomic landscape of current and future environmental adaptation. *Ecology Letters* **18**, 1-16.

Guillot G, Estoup A, Mortier F, Cosson JF (2005a) A spatial statistical model for landscape genetics. *Genetics* **170**, 1261-1280.

Guillot G, Mortier F, Estoup A (2005b) Geneland: a computer package for landscape genetics. *Molecular Ecology Notes* **5**, 712-715.

Guillot G, Santos F, Estoup A (2008) Analysing georeferenced population genetics data with Geneland: a new algorithm to deal with null alleles and a friendly graphical user interface. *Bioinformatics* **24**, 1406-1407.

Hebert PDN, Stoeckle MY, Zemlak TS, Francis CM (2004) Identification of birds through DNA barcodes. *PLoS Biology* **2**, 1657-1663.

Larkin MA, Blackshields G, Brown NP*, et al.* (2007) Clustal W and Clustal X version 2.0. *Bioinformatics* **23**, 2947-2948.

Long DG, Drinkwater MR, Holt B, Saatchi S, Bertoia C (2001) Global ice and land climate studies using scatterometer image data. *EOS Transactions AGU* **82**, 503.

Okonechnikov K, Golosova O, Fursov M, UGENE-Team (2012) Unipro UGENE: a unified bioinformatics toolkit. *Bioinformatics* **28**, 1166-1167.

Pritchard JK, Stephens M, Donnelly P (2000) Inference of population structure using multilocus genotype data. *Genetics* **155**, 945-959.

Schuelke M (2000) An economic method for the fluorescent labeling of PCR fragments. *Nature Biotechnology* **18**, 233-234.

Tamura K, Stecher G, Peterson D, Filipski A, Kumar S (2013) MEGA6: Molecular Evolutionary Genetics Analysis Version 6.0. *Molecular Biology and Evolution* **30**, 2725-2729.

Thompson JD, Gibson TJ, Plewniak F, Jeanmougin F, Higgins DG (1997) The ClustalX windows interface: flexible strategies for multiple sequence alignment aided by quality analysis tools. *Nucleic Acids Research* **25**, 4876-4882.
